# Antibiotic Susceptibility of Microorganisms Grown in Tracheal Aspirate Cultures of Pediatric Intensive Care Patients

**DOI:** 10.7759/cureus.26934

**Published:** 2022-07-17

**Authors:** Özhan Orhan, Kamil Yılmaz, Ayfer Gözü Pirinççioğlu, Murat Solmaz, Ferhat Karakoç

**Affiliations:** 1 Pediatrics, Kızıltepe State Hospital, Mardin, TUR; 2 Department of Pediatrics, Dicle University Hospital, Diyarbakır, TUR; 3 Pediatric Medicine, Kozluk Devlet Hastanesi, Batman, TUR; 4 Pediatric Medicine, Ergani Devlet Hastanesi, Diyarbakır, TUR

**Keywords:** intensive care unit, pediatric, empirical therapy, drug resistance, tracheal aspirate

## Abstract

Background

Microorganisms proliferating in the hospital setting cause infections with high morbidity and mortality rates. In intensive care units (ICUs), the rates of antibiotic resistance and microorganisms grown in cultures may vary by time period. Antibiotic sensitivity must be known for a correct empirical treatment approach. This study aimed to investigate the distribution and antibiotic resistance profiles of pathogenic microorganisms isolated from tracheal aspirate samples in the ICU.

Methodology

This study enrolled 100 tracheostomized patients aged one month to 18 years, regardless of gender, who were followed in the ICU of Dicle University for more than 72 hours. Medical data were retrospectively evaluated from the medical records. Care was taken to collect samples before changing antibiotics. Antibiotherapy was continued until after culture antibiogram results were obtained, or empirical antibiotic therapy was started by giving consideration to the potential source in patients with a suspected infection.

Results

An analysis of the tracheal aspirate culture samples of the patients showed that *Pseudomonas aeruginosa *(54%), *Acinetobacter baumannii *(16%), and *Staphylococcus aureus *(8%) were the most common pathogens. An analysis of the culture antibiogram results of the tracheal aspirate samples obtained from the entire study population showed that *P. aeruginosa* was 100% resistant against vancomycin, clindamycin, and teicoplanin, but highly sensitive to colistin and amikacin. *A. baumannii* was highly resistant to almost all antibiotics but showed no resistance against colistin. Carbapenems being frequently preferred for cases where empirical therapy should be initiated for ICU infections can be one of the reasons for a high carbapenem resistance rate in our hospital.

Conclusions

We believe that starting empirical therapy with colistin when infections caused by *Pseudomonas* and *Acinetobacter *are suspected may be an appropriate initial therapy until culture antibiogram results become available. Microbiological data are crucial for a correct empirical treatment approach. In this way, intensive antibiotic usage and subsequent high antibiotic resistance can be adequately controlled.

## Introduction

Nosocomial infections are defined as infections that are neither present at the time of hospital presentation nor in the incubation period at hospital admission [1]. A great majority of nosocomial infections are seen in intensive care units (ICUs). Despite the advancements in medicine, nosocomial infections remain an important health problem worldwide [2].

Pediatric intensive care units (PICU), where nosocomial infections are common, are a setting where broad-spectrum antibiotics are used because of the existence of resistant pathogens [3].

In ICUs, invasive procedures such as mechanical ventilation, tracheostomy, and catheter placement, as well as the duration of ICU stay, are the main factors associated with infections caused by resistant pathogens [3].

Pneumonia is the leading cause of death among children aged less than five years worldwide. Approximately 1.4 million of a total of 7.6 million deaths in the pediatric population in 2010 occurred due to pneumonia [4]. Pneumonia is one of the most common nosocomial infections. Clinical and radiological findings may not be necessarily sensitive or specific enough for diagnosing pneumonia. Thus, gram staining and culture studies of lower respiratory tract samples, such as endotracheal aspirate (ETA) obtained with a protected specimen brush and bronchoalveolar lavage (BAL), aid in diagnosis and management [5]. It is important to accurately detect the etiological agent and promptly start antimicrobial therapy. A delay of four to eight hours during treatment has been shown to increase mortality [5,6]. Hence, empirical antibiotic therapy is usually started by the clinician without waiting for laboratory findings.

Bloodstream infections (BSIs) are among the leading causes of mortality and morbidity worldwide. Blood cultures remain the main and the most sensitive method for diagnosis. Early detection and identification of the causative microorganisms with blood cultures, determination of antibiotic sensitivity, and administration of the appropriate therapy are important steps that reduce mortality. Automatic blood culture systems that detect microorganisms better and faster are currently the most preferred method for culturing blood samples [7,8]. Several differences regarding microorganisms, antibiotic resistance rates, and the distribution of antibiotherapy can be observed between hospitals or even at different time points in the same ICU. Factors detected in those units and antibiotic sensitivities should be determined at regular intervals, and treatment protocols should be updated according to the follow-up results.

This study aimed to determine the distribution of pathogenic microorganisms isolated from tracheal aspirate samples and their antibiotic resistance profiles in the ICU.

## Materials and methods

This study enrolled 100 tracheotomized patients aged one month to 18 years, regardless of gender, who were admitted to the Department of Pediatrics Intensive Care Unit, Dicle University, between January 2016 and January 2017 and monitored for more than 72 hours. The study was approved by the local institutional review board of clinical investigation at Dicle University and Turkey Health Minister (approval number: 120). The patients’ data were retrospectively evaluated using their medical records.

Deep tracheal aspirate or sputum samples (via closed system aspiration) and urine samples were collected, and axillary, rectal, and nasal swabs were taken for surveillance at the time of ICU admission. At least two blood cultures were taken in patients with a body temperature of >38.3°C. Two blood cultures were taken from patients with suspected catheter infection, one from peripheral circulation and the other from the central catheter. Additionally, the tip of a removed central catheter was sent for culture. Urine samples for urine cultures were obtained from a newly placed catheter. Additionally, deep tracheal aspirate samples were collected via closed system aspiration once per week during the ICU stay or when ventilator-associated pneumonia was suspected. BAL was performed when needed, and care was taken to collect samples before changing the antibiotics. By evaluating culture results in conjunction with the clinical signs of infection, we aimed to determine a patient’s ongoing infection or colonization status at the time of admission, as well as the microorganisms colonizing the patients during their ICU stay. The current antibiotherapy was continued until culture antibiogram results were obtained, while empirical antibiotic therapy was started considering the potential source in the case of suspected infection. Proliferation in the tracheal aspirate samples collected at the time of ICU admission was defined as pneumonia if it was supported by clinical and laboratory tests. The absence of proliferation in the first culture followed by proliferation in the subsequent cultures was defined as ventilator-associated pneumonia. In cases where addition or change in antibiotics was considered, the change was done empirically after obtaining the required culture sample.

Patients without any proliferation of causative microorganisms in the cultures were excluded. The patients’ data were obtained from the hospital database and ICU surveillance data.

Statistical analysis

Statistical analysis of the study data was performed using the SPSS version 26 software package (IBM Corp., Armonk, NY, USA). Descriptive statistics included number (n) and percentage (%). Independent groups were compared using Student’s t-test. Inter-group differences regarding frequencies were tested using chi-square tests. All hypotheses were tested in a two-sided manner, and p ≤ 0.05 was considered statistically significant.

## Results

Of the study participants, 66% were girls and 34% were boys. The mean age of the study population was 29.04 ± 29.386 (minimum = 3, maximum = 164) months. Fever was the most common sign in 66% of patients treated in the ICU. Overall, 50% of the cases had a history of hospital stay for 201 days or longer, 18% had a stay of 0-50 days, 14% had a stay of 51-100 days, 11% had a stay of 151-200 days, and 7% had a stay of 101-150 days.

Tracheal aspirate culture analysis of the patients most commonly showed *Pseudomonas aeruginosa* (54%), *Acinetobacter baumannii* (16%), and *Staphylococcus aureus* (8%) (Table 1).

**Table 1:** Microorganisms grown in the tracheal aspirate samples.

Microorganism	n (N = 100)	%
Pseudomonas aeruginosa	54	54
Acinetobacter baumannii	16	16
Staphylococcus aureus	8	8
Klebsiella pneumoniae	7	7
Serratia marcescens	7	7
Methicillin-resistant *Staphylococcus aureus*	2	2
Escherichia coli	1	1
Others	5	5

In total, 23 (23%) of the patients died despite all treatments. The most frequently isolated microorganisms in tracheal aspirate samples from the deceased patients were *P. aeruginosa* (52.1%) and *S. aureus* (17.3%). Other microorganisms isolated are shown in Table 2.

**Table 2:** Results obtained in tracheal aspirate samples.

	n (N = 23)	%
Pseudomonas aeruginosa	12	52.1
Staphylococcus aureus	4	17.3
Acinetobacter baumannii	3	13
Serratia marcescens	2	8.6
Klebsiella pneumoniae	1	4.3
Others	1	4.3
Methicillin-resistant *Staphylococcus aureus*	0	0
Escherichia coli	0	0

An analysis of the culture antibiogram results of the tracheal aspirate samples of the whole study population revealed that* P. aeruginosa* showed 100% resistance against vancomycin, clindamycin, and teicoplanin but was highly sensitive to colistin and amikacin. *A. baumannii*, on the other hand, was highly resistant to all antibiotics but showed no resistance against colimycin. Antibiogram resistance results of the other microorganisms are shown in Table 3.

**Table 3:** Antibiotic resistance status of microorganisms grown in the tracheal aspirate samples. AK: amikacin; VAN: vancomycin; CRO: ceftriaxone; CTX: cefotaxime; CAZ: ceftazidime; TZP: tazobactam; LNZ: linezolid; MEM: meropenem; IMI: imipenem; SXT: trimethoprim/sulfamethoxazole; CLI: clindamycin; CIP: ciprofloxacin; KOL: colomycin; SUL: sulperazone; PEN: penicillin G; TEI: teicoplanin

	n	AK n (%)	VAN n (%)	CRO n (%)	CTX n (%)	CAZ n (%)	TZP n (%)	LNZ n (%)	MEM n (%)	IMI n (%)	SXT n (%)	CLI n (%)	CIP n (%)	KOL n (%)	SUL n (%)	PEN n (%)	TEI n (%)
Pseudomonas aeruginosa	54	9 (16.7)	54 (100)	51 (94.4)	50 (92.6)	22 (40.7)	18 (33.3)	52 (96.3)	37 (68.5)	43 (79.6)	39 (72.2)	54 (100)	38 (70.4)	2 (3.7)	36 (66.7)	52 (96.3)	54 (100)
Acinetobacter baumannii	16	15 (93.8)	16 (100)	16 (100)	16 (100)	16 (100)	16 (100)	16 (100)	16 (100)	15 (100)	13 (81.2)	16 (100)	16 (100)	0 (0)	16 (100)	16 (100)	16 (100)
Staphylococcus aureus	8	1 (12.5)	0 (0)	4 (50)	4 (50)	4 (50)	1 (12.5)	0 (0)	1 (12.5)	1 (12.5)	2 (25.0)	0 (0)	0 (0)	1 (12.5)	4 (50)	7 (87.5)	0 (0)
Serratia	7	1 (14.3)	7 (100)	2 (28.6)	5 (71.4)	1 (14.3)	2 (28.6)	7 (100)	3 (42.9)	4 (57.1)	2 (28.6)	6 (85.7)	4 (57.1)	6 (85.7)	3 (42.9)	7 (100)	7 (100)
Klebsiella	7	0 (0)	7 (100)	4 (66.7)	5 (71.4)	5 (71.4)	4 (57.1)	7 (100)	2 (28.6)	1 (14.3)	4 (57.1)	6 (85.7)	3 (42.9)	0 (0)	5 (71.4)	7 (100)	7 (100)
Escherichia coli	1	0 (0)	1 (100)	1 (100)	1 (100)	1 (100)	1 (100)	1 (100)	0 (0)	0 (0)	0 (0)	1 (100)	1 (100)	0 (0)	1 (100)	1 (100)	1 (100)
Other	7	5 (100)	9 (16.7)	4 (80.0)	3 (75)	4 (80)	4 (80)	3 (60.0)	4 (80)	4 (0)	2 (40)	4 (80)	4 (80)	4 (80)	4 (80)	4 (80)	3 (60)
Total	100	31 (31)	88 (88)	82 (82)	84 (84)	53 (53)	46 (46)	86 (86)	63 (63)	68 (68.7)	62 (62)	87 (87)	66 (66)	13 (13)	69 (69)	94 (94)	88 (88)

## Discussion

Globally, 30% of all nosocomial infections are seen in ICUs. These infections are an important cause of mortality and morbidity [9]. The most common nosocomial infections include surgical site infections, BSIs, urinary tract infections [10], respiratory tract infections, gastroenteritis, pneumonia, meningitis, and other soft tissue infections [11]. Long hospital stays, invasive procedures, low birth weight, total parenteral feeding, and congenital anomalies increase the risk of infection [12]. The immature immune system in newborns also facilitates healthcare-associated infections [13]. While gram-positive bacteria are reported in nosocomial infections in developed countries, gram-negative bacteria have been reported in developing countries [14-16].

Hospitalization in ICUs forms a basis for infection due to invasive procedures breaking the natural barrier, which increases in number as the duration of ICU stay is prolonged [17]. Because resistant microorganisms found in the hospital flora are the causative microorganisms, healthcare-associated infections (HCAIs) are difficult to treat and typically require long-term treatment with broad-spectrum and expensive antimicrobials [18]. HCAIs cause prolonged hospitalization, increased healthcare costs, morbidity, mortality, and workforce and productivity losses [18-21]. A prospective matched case-control study conducted in Argentina found that 142 individuals with central catheter-associated bloodstream infections (CA-BSIs) had a longer hospitalization duration by an average of 11.9 days, an additional cost of 4,888.42 dollars, and 24.6% higher mortality compared with the controls who were free of infections [22]. Similarly, a study conducted in Mexico reported that HCAIs caused an extra hospitalization duration of an average of 6.05 days, an extra cost of 8,326 dollars, and 20% higher mortality [23]. Lower respiratory tract infections are among the most common nosocomial infectious foci seen in ICUs (12). Gram-negative, non-fermentative bacteria such as *P. aeruginosa* and *A. baumannii*, which are associated with high mortality and morbidity rates, are among the most common etiological agents causing these infections. According to the antimicrobial surveillance program (SENTRY), the gram-negative microorganisms that are most commonly isolated in ICUs include *Escherichia coli*, *Klebsiella pneumoniae*, *P. aeruginosa*, *Enterobacter *spp, *Serratia *spp, *Hemophilus influenza*, *A. baumannii*, and *Proteus mirabilis*. In addition, two studies investigating microorganisms grown in tracheal aspirate cultures in ICUs in Turkey found that *A. baumannii* and *P. aeruginosa* were the most common microbiological agents [13,15]. Studies on the carbapenem resistance of *A. baumannii* isolates grown from the samples sent from Turkey as part of the SENTRY program found sensitivity rates of 80.4% and 71.7% for imipenem and meropenem, respectively, as of 2000. Özünel et al. [17] reported that imipenem resistance was 86% among *Acinetobacter *strains grown in ETA cultures between 2012 and 2013. Aydemir et al. [18] found a rate of 93.3% for imipenem resistance among *Acinetobacter *strains grown in ETA cultures between 2015 and 2016. *S. aureus* constituted 28% of all nosocomial and ventilator-associated pneumonia (VAP) agents in the SENTRY Antimicrobial Research Program. Aydemir et al. [18] found methicillin resistance in 30% of *S. aureus* strains. According to our study findings, *P. aeruginosa* (54%), *A. baumannii* (16%), and *S. aureus* (8) were the most commonly isolated microorganisms.

*P. aeruginosa* had a mean percentage of resistance of 70% against the carbapenem group but was highly sensitive to colimycin and aminoglycosides. *A. baumannii*, on the other hand, was 100% resistant against the carbapenem group and 94% against amikacin while it showed no resistance against colimycin.

The type and capacity of ICUs, use of different antibiotic treatment protocols, hospital or community-acquired bacteremia, and the number and characteristics of patients included in the studies are some of the factors associated with the differences noted between centers.

The factors associated with high rates of resistance among microorganisms grown in the ETA cultures of our patients hospitalized in our ICUs include intensive invasive treatments, long hospital stays, and administration of broad-spectrum antibiotics instead of limited antibiotic administration.

The frequent use of carbapenems in cases where empirical treatment should be initiated for ICU infections may be considered as one of the reasons for higher carbapenem resistance rates in our hospital. Therefore, when infections caused by *Pseudomonas *and *Acinetobacter *are suspected, we believe that beginning empirical therapy with colimycin may be an appropriate starting treatment until culture antibiogram results become available.

Because methicillin-resistant *Staphylococcus aureus* was detected in only two patients in our study, we thought that the evaluation of antibiotic resistance against the relevant microorganism would not yield accurate results. This was one of the limitations of our study.

## Conclusions

Despite the advancements in the treatment and prevention of VAP, it remains an important cause of morbidity and mortality in nosocomial infections. Each hospital, using knowing its own microbiological flora and resistance pattern, can reduce mortality by instituting early and correct treatment. Microbiological data are crucial for a correct empirical treatment approach. In this way, intensive use of antibiotics and subsequent high resistance rates will be adequately controlled.
